# Cerebellar Progressive Multifocal Leukoencephalopathy Mimicking Anti-Yo-Antibody-Associated Rapidly Progressive Cerebellar Syndrome

**DOI:** 10.3390/neurolint15030059

**Published:** 2023-07-26

**Authors:** Takayoshi Akimoto, Makoto Hara, Satoshi Hirose, Kazuo Nakamichi, Hideto Nakajima

**Affiliations:** 1 Division of Neurology, Department of Medicine, Nihon University School of Medicine, Tokyo 173-8610, Japan; 2 Department of Virology 1, National Institute of Infectious Diseases, Tokyo 162-8640, Japan

**Keywords:** progressive multifocal leukoencephalopathy, anti-Yo antibody, rapidly progressive cerebellar syndrome, systemic lupus erythematosus, cerebellar ataxia

## Abstract

A 58-year-old woman with a history of systemic lupus erythematosus (SLE) who was taking prednisolone and mycophenolate mofetil presented with gait disturbances that progressively worsened over a period of 3 months. Her blood test and cerebrospinal fluid (CSF) examination results did not indicate active SLE. Initial brain magnetic resonance imaging (MRI) revealed a small spotty lesion in the left cerebellar peduncle. The clinical course was consistent with rapidly progressive cerebellar syndrome (RPCS), which sometimes involves neuronal antibodies. The line blot assay detected anti-Yo antibodies, but no malignancy was found. Immunohistological techniques using rat brain sections yielded a negative result for anti-Yo antibodies. The second MRI revealed a focal lesion and surrounding spotty lesion in the left cerebellar peduncle, which was consistent with the punctate pattern observed in progressive multifocal leukoencephalopathy (PML). The CSF JCV-DNA test indicated the presence of cerebellar PML. Immunosuppressants were reduced, and mefloquine and mirtazapine were initiated. After approximately 2 years and 1 month, the CSF JCV-DNA results became negative. Cerebellar PML may exhibit a clinical course that is consistent with RPCS. The punctate pattern should be recognized as an early manifestation of PML. The CSF JCV-DNA copy number may serve as a useful indicator of PML stabilization.

## 1. Introduction

Progressive multifocal leukoencephalopathy (PML) is a demyelinating central nervous system disease caused by John Cunningham virus (JCV) disease [1,2]. JCV is a widely spread virus, with a worldwide antibody prevalence of >50% of the adult population [2,3]. JCV is a double-stranded DNA virus classified within the Polyomavirus family. It consists of an archetype, which possesses a stable genome and is nonpathogenic, and a prototype, which exhibits mutations characterized by DNA sequence deletions and/or duplications in the noncoding control region (NCCR) [1,2,4]. The prototype JCV causes reactivation and replication in oligodendrocytes, causing central demyelination [2,4]. Immune responses against JCV infection involve antibody, CD8+ cytotoxic T cell, and CD4+ T lymphocyte production [5,6]. Therefore, PML develops with immunosuppression such as human immunodeficiency virus (HIV) infection, lymphoma, leukemia, systemic lupus erythematosus (SLE), and organ transplantation [1,2,3,4]. Some PML cases have been attributed to the use of antibody drugs such as natalizumab, rituximab, and efalizumab [1,2,4,7,8]. PML typically occurs after a subacute course and presents with various symptoms, including cognitive impairment, motor dysfunction, gait disturbance, language impairment, visual field disturbance, and sensory disturbance [3,4,9,10]. Specific symptoms vary depending on lesion location, emphasizing the significance of neuroimaging, particularly magnetic resonance imaging (MRI). PML lesions exhibit high signal intensity on T2 and fluid-attenuated inversion recovery (FLAIR) sequences, low signal intensity on T1, and occasional contrast enhancement, extending from white matter to subcortical white matter [3,11]. PML diagnosis relies on clinical symptomatology, imaging findings, and confirmation of the presence of JCV through JCV-DNA detection in cerebrospinal fluid and/or brain biopsy [3,4]. Therefore, PML should be considered a potential cause when immunosuppressed patients present with subacute central nervous system symptoms. PML more frequently appears in supra-tentorial lesions than in sub-tentorial lesions [11]. Cerebellar involvement in PML may present with typical cerebellar symptoms, including dizziness, vertigo, nausea, limb ataxia or clumsiness, gait disturbances, and speech difficulties [12,13,14,15,16,17,18,19,20].

A variety of factors other than PML, including immune-mediated, infectious, degenerative, and deficiency diseases, as well as drug or alcohol abuse, cause progressive cerebellar ataxia [21]. In particular, a cerebellar syndrome interfering with daily life within 3 months is known as rapidly progressive cerebellar syndrome (RPCS), which is sometimes associated with anti-Yo antibodies [22]. This case report describes a patient who initially tested positive for anti-Yo antibodies by line blot assay, but the immunohistological test was negative, ultimately indicating a cerebellar PML diagnosis. Distinguishing cerebellar PML from anti-Yo-antibody-associated RPCS was difficult based on the clinical symptoms. Here, we present the interpretation of anti-Yo-antibody-positive cases and the characteristics of MRI findings that should raise suspicion for early cerebellar PML.

## 2. Case Presentation

A 57-year-old woman presented with dizziness and progressive gait disturbance for approximately three months. The patient was diagnosed with SLE eight years before the visit and received 5 mg/day of prednisolone (PSL) and 1500 mg/day of mycophenolate mofetil (MMF). The patient has received follow-up treatment by a rheumatologist since disease onset, and her SLE symptoms were well-controlled for eight years. Except for the SLE, the patient’s medical history was unremarkable. The patient was a homemaker with no exposure to toxins and no travel abroad and denied any dietary deviations, drinking, or smoking. There was no family history of neurological diseases, including neurodegenerative disorders.

Upon initial examination (day 1), her body temperature was 36.4 °C. Consciousness, blood pressure, heart rate, and oxygen saturation were normal. No scleritis, arthritis, or rash were observed. The lung and cardiac examinations were normal. The patient’s speech, eye movement, and facial movements and sensations were normal. Muscle strength, muscle tone, and tactile, pain, and vibratory sensations in the extremities were normal. The finger–nose–finger test was normal, but the heel-to-shin test revealed mild bilateral ataxia. Biceps and patellar tendon reflexes were bilaterally hyperactive, but pathological reflexes were unremarkable. The patient could stand, but exhibited a wide-base gait and was unable to walk alone, requiring assistance. The Romberg test was negative, and no signs of autonomic dysfunction or meningeal irritation were detected.

**Figure 1:**
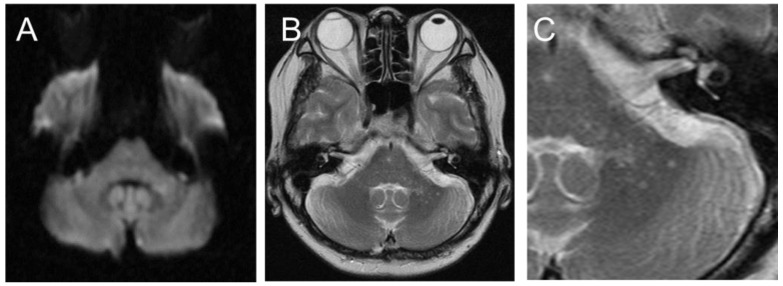
Initial brain magnetic resonance imaging (MRI) showed a small, high-intensity lesion in the left cerebellar peduncle on diffusion-weighted imaging (**A**). On T2-weighted imaging (**B**), the lesion appeared to be a cluster of small spots ((**C**): enlarged image of (**B**)).

Blood tests revealed a high titer of antinuclear antibodies (1:640), which was similar to last year. Otherwise, no other findings indicated increased SLE activity. Her vitamin B1 levels were 22 ng/mL (normal range: 24 ng/mL and above), and her vitamin B12 levels were 114 pg/mL (normal range: 180 pg/mL and above). The HIV antibody was negative. Furthermore, glutamic acid decarboxylase, thyroid peroxidase, and thyroglobulin were negative. Evaluation of her CSF revealed no elevation in cell count (1/mm^3^), total protein level (25 mg/dL), IgG index (0.44), or interleukin-6 (3.8 pg/mL). Brain MRI on day 7 showed no cerebellar atrophy, and small spotty hyperintense lesions without mass effect were observed in the left cerebellar peduncle on diffusion-weighted and T2-weighted images (Figure 1). The hyperintense lesions did not show a low signal in the apparent diffusion coefficient map, and no gadolinium enhancement was observed.

**Figure 2:**
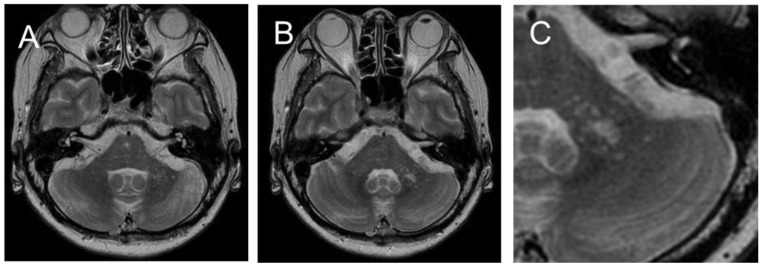
MRI on day 39 revealed increasing spotty lesions in the left peduncle that became centrally clustered, with a spreading spotty lesion surrounding the central area (**A**,**B**). ((**C**): Enlarged image of (**B**)). No cerebellar cortex atrophy was observed.

Vitamin B12 deficiency was suspected to play a role in the symptoms, and transvenous vitamin supplementation was initiated. Although the vitamin B12 levels sufficiently increased, the patient’s gait disturbance gradually worsened. A serum line blot assay test for 12 paraneoplastic antibodies (i.e., Hu, Yo, CV2, Ri, Ma2/Ta, GAD65, amphiphysin, recoverin, SOX1, titin, zic4, and Tr, EUROLINE, Euroimmun, Lübeck, Germany) was performed, and the anti-Yo antibody was positive. Contrast-enhanced computed tomography, pelvic MRI, mammary echocardiography, gallium scintigraphy, fecal occult blood test, and upper gastrointestinal endoscopy failed to detect any malignancies. After 3 weeks, the patient became bedridden due to dizziness and nausea and developed mild ataxia in the upper extremities. The patient had difficulty taking MMF capsules internally; thus, MMF was discontinued, and the PSL dose was increased to 15 mg/day. Brain MRI on day 39 showed a focal lesion and surrounding spotty lesions in the left cerebellar peduncle. The cerebellar atrophy was not remarkable (Figure 2).

Progressive cerebellar ataxia which significantly affected daily life within three months was consistent with RPCS involving the anti-Yo antibody [22]. On day 67, positron emission computed tomography was performed in another hospital for advanced malignancy screening, but no evidence of malignancy was found. An in-house tissue-based assay (TBA) was performed using rat frozen cerebellar sections, which yielded a negative result for the anti-Yo antibody (Supplementary Figure S1).

In natalizumab-related and SLE-related PML, small punctate lesions occur as early PML imaging features, called the “punctate pattern” or “milky way” [11,23,24,25,26]. The spotty lesions observed in the left peduncle in our patient indicated PML. Real-time PCR was used to measure the CSF JCV-DNA [27]. While waiting for the examination results, ataxia of the extremities and trunk progressively worsened, causing the patient to remain bedridden. Although physical examination and laboratory results did not indicate active SLE, steroid pulse therapy and intravenous immunoglobulin therapy (IVIg) were started based on the possibility of central nervous system lupus from day 70, and PSL of 15 mg/day was continued.

**Figure 3:**
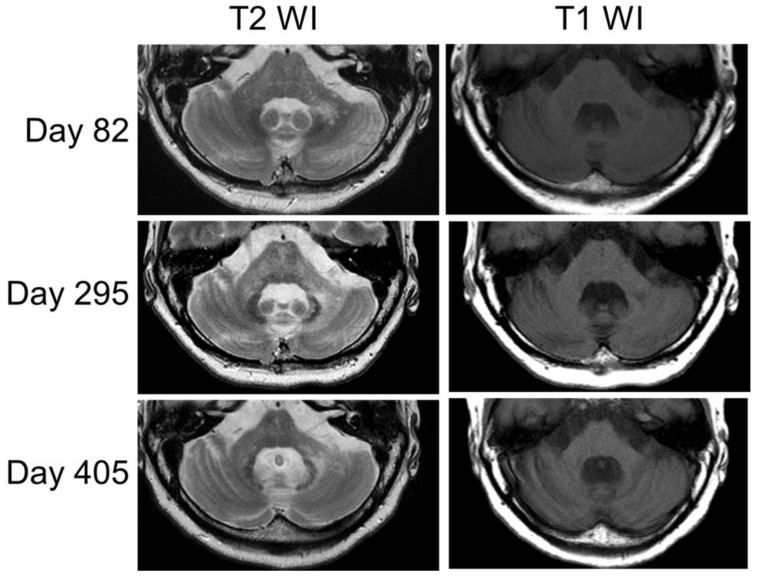
The MRI findings changed over time (days 82, 295, and 405). The left peduncle lesion appeared enlarged on the T2-weighted images, and with this enlargement, atrophy became prominent in both the cerebellum and brainstem on the T1-weighted images.

On day 76, JC virus DNA at 15,870 copies/mL was detected in the patient’s CSF. Additionally, PCR typing was performed, targeting a DNA sequence within the viral genome (region D of the NCCR) retained in the archetype JCV but often missing in the prototype JCV [28]. The JCV detected in the patient’s CSF lacked this region and was identified as a prototype with a mutation. Therefore, the patient was diagnosed with cerebellar PML. The scale for the assessment and rating of the ataxia (SARA) score [29] was 25.5. In consultation with a rheumatologist, the PSL dose was reduced. In addition, 15 mg/day of mirtazapine and 275 mg/week of mefloquine were initiated; off-label use of these drugs was approved by the pharmaceutical affairs committee. On day 82, the T2 hyperintense lesion in the left cerebellar peduncle extended to the brainstem (Figure 3).

On day 114, the SARA score was 27.0. Since that point, the patient’s clinical symptoms neither worsened nor improved (the SARA score remained at 27.0). On day 193, the CSF JCV-DNA was retested and was found to decrease to 30 copies/mL. On day 295, MRI revealed T2 high-intensity spreading to the pons and right peduncle, and the pontine and cerebellar cortex atrophy progressed (Figure 3). Despite the PSL decrease (6 mg/day), the JCV-DNA remained at 43 copies/mL on day 300. On day 358, the patient was diagnosed with an SLE flare-up based on generalized myalgia, arthralgia, decreased CH50, and increased anti-ds DNA; thus, the PSL dose was temporarily increased. On day 405, the MRI showed pontine and cerebellar cortex atrophy progression (Figure 3); however, no considerable change was observed in the patient’s clinical presentation.

On day 470, the patient was discharged home in a reclining wheelchair with sustained seating and was put on a soft meal. The patient continued to regularly visit our hospital in the reclining wheelchair. On day 778, the CSF JCV-DNA was below the detection limit, and mefloquine and mirtazapine were discontinued. MRI on day 834 did not indicate brainstem cerebellar atrophy progression, and there was no deterioration in the patient’s neurological findings.

## 3. Discussion

A middle-aged woman with a history of SLE presented to our hospital with a clinical course consistent with RPCS. Her MRI results revealed a punctate pattern, which increased suspicions of cerebellar PML. The diagnosis was confirmed by measuring the JCV-DNA in the patient’s CSF. The immunosuppressants were reduced, and mefloquine and mirtazapine were initiated. The CSF JCV-DNA decreased below the sensitivity level, cerebellar atrophy ceased, and long-term survival was achieved. Commercial line blot assays often give false-positive results in patients without tumors [20]; thus, we ruled out anti-Yo-antibody-associated RPCS in this case based on the long-term follow up and TBA-negative results. Based on the initial symptoms and clinical course, distinguishing cerebellar PML from anti-Yo-antibody-associated RPCS was difficult.

Progressive cerebellar ataxia that interferes with daily life within 3 months is referred to as RPCS [22]. RPCS is associated with the anti-Yo antibody, anti-Tr antibody [22], anti-GAD antibody [30,31], or SLE [32,33]. In this case, the line blot assay was positive for anti-Yo antibodies, which was considered the cause of RPCS. However, commercial line blot assays for the anti-Yo antibody have low specificity [22,34]. These assays are targeted against CDR2; however, recent studies have demonstrated that the actual target of the anti-Yo antibody is CDR2L, which has only 45% homology with CDR2 [34,35]. Therefore, false-positive results may be obtained when only testing for CDR2 using line blot assays, which are not specific to RPCS. For anti-Yo-antibody-positive cases that do not conform to paraneoplastic neurological syndrome, the line blot assay and immunohistology results are concordant in only 8% (1/13) of cases [34]. Therefore, it is important to confirm the presence of positive anti-Yo antibodies through immunohistological tests such as TBA, especially in cases without malignancy [22,34].

There have been reported cases of SLE patients with a clinical course of RPCS [32,33]. In these reports of SLE-related RPCS, SLE activity was elevated when RPCS was present [32,33]. The pathogenesis of SLE-associated RPCS was suggested to be due to autoimmunity, cerebrovascular disease, or angiogenic edema, and the clinical symptoms were improved by immunotherapy [32,33]. In the present case, there were no signs of active SLE, and the patient’s clinical symptoms did not improve with steroid pulse therapy and IVIg. This suggests that SLE was unlikely to have contributed to the RPCS in this case. If SLE-associated RPCS is suspected, disease activity and response to immunotherapy should be confirmed.

SLE is considered as a risk factor of PML [36]; therapeutic glucocorticoids and MMF are also implicated in drug-related PML [8]. The neurological symptoms of PML are often cerebrum-related (cognitive dysfunction, language impairment, motor paralysis, etc.) [3], and a multicenter study of PML images reported that supra-tentorial lesions are more common than sub-tentorial lesions (supra-tentorial: 69.4%, sub-tentorial: 11.1%, both: 19.4%) [11]. Determining the imaging findings that should raise the suspicion of PML in PML-risk cases with a course of RPCS is important because the differentiation of RPCS varies widely. The MRI findings of progressed cerebellar PML were T2 and FLAIR hyperintense lesions extending from the cerebellar peduncle to the periventricular area of the fourth ventricle [12,14,15,16,19,20,37,38]. A punctate pattern characterizes natalizumab-associated PML [11,23,26], SLE-associated PML [24], and immune reconstitution inflammatory syndrome associated with PML [25,26]. In the present case, the focal lesion and surrounding spotty lesions in the left cerebellar peduncle were noticeable on day 39; however, the first MRI on day 7 also revealed a small spotty lesion. After the punctate pattern was observed, the lesion expanded along the fourth ventricle with time, and cerebellar and brainstem atrophy became evident. It is noteworthy that the punctate pattern in the MRI findings is an early manifestation of PML.

The treatment of PML often involves resolution of the immunosuppressive condition and discontinuation of the causative agent [4,12,14,15,16,39]. Combination antiretroviral therapy against HIV [4,39] is used for HIV-related PML, and plasma exchange is used for natalizumab-related PML [40]. However, SLE-related PML has no specific treatment. Mirtazapine, which is a noradrenergic and specific serotonergic antidepressant, has prevented the virus from binding to oligodendrocytes, and mefloquine, which is an antimalarial agent, has exhibited antiviral activity in vitro and may be used [4,12,16,19,41]. The PSL dose was reduced after consultation with a rheumatologist and after MMF discontinuation in the present case. However, it was difficult to assess the PML activity based on the time course of clinical symptoms. Mainly in HIV-associated PML, CSF JCV-DNA levels have been associated with PML stability [39,41]; therefore, the JCV-DNA copy number is thought to be an indicator of PML activity. Therefore, we measured the JCV-DNA copy number over time, and the JCV-DNA copy number decreased. Eventually, the JCV-DNA level decreased below the detection limit, and the patient achieved long-term survival.

The association between JCV-DNA copy number and background disease, treatment, and outcome was investigated by collecting case reports of SLE-associated cerebellar PML. Table 1 presents cases of SLE-associated cerebellar PML [12,13,14,15,16,17,18,19,20,37,38].

Of the 12 patients, 10, including ours, were women [12,13,14,15,17,18,19,38]. In addition to SLE, two patients had rheumatoid arthritis [14,15], and one had a history of lymphoma [19] and hemophagocytic syndrome [13]. Corticosteroids were used in all patients for whom information was available, and other immunosuppressants used concomitantly were methotrexate in four patients [13,14,16,38]; rituximab in three [13,14,19]; MMF [19], etanercept [14,15], and azathioprine [12,13] in two; and cyclophosphamide [13], cyclosporine [20], tacrolimus [16], bendamustine [19], hydroxychloroquine [15], tocilizumab [14], and thalidomide in one patient. Eight patients were diagnosed with PML by spinal fluid specimens [13,14,15,16,20,38], two by biopsy [17,37], one by spinal fluid and biopsy [19], and one by autopsy [18]. The CSF JCV copy number ranged from 700 to 67,000 copies/mL [12,15,38]. The treatments included mefloquine in five patients [12,14,16,19], mirtazapine in four [12,16,19], cidofovir in three [12,13,20], plasmapheresis (plasma exchange, double filtration plasmapheresis one each) in two [14,15], cytarabine in two [12,15], paroxetine in one [14], and interferon in one [18]. Additionally, the dose of immunosuppressants was reduced in all six patients [12,14,15,16,38], in which a change in SLE treatment was mentioned (not shown in Table 1). The JCV-DNA was retested in five patients and turned negative [14,16,20] in all except one [12]. The contribution of JCV-DNA negativity to survival demonstrated that three of the four negative cases (75%) [14,16,20] were alive. This rate is higher than the survival rate in the review [36], but the number of cases is small, and more cases need to be accumulated. Of the 11 patients for which an outcome was shown, 7 (63.6%) died during the follow-up period of 3 to 36 months [12,15,17,18,19,20,38]. In one of the patients who died, the JCV-DNA levels decreased below the detection limit [20]. In the case report, the cause of death was kidney failure with elevated urinary protein, suggesting the involvement of SLE flare-up in the death [20]. Therefore, in patients with SLE-associated PML, the dose of immunosuppressants should be carefully assessed and reduced while evaluating disease activity.

## 4. Conclusions

Cerebellar PML can present a clinical course consistent with RPCS. If positive anti-Yo antibodies are detected by line blot assay, additional immunohistological test is necessary, particularly in cases without malignancies. Early identification of PML, which is characterized by focal lesions and surrounding small spotty lesions called a “punctate pattern,” in MRI findings is necessary. Although cerebellar PML has poor prognosis, the JCV-DNA copy number may serve as an indicator of PML stability.

## Figures and Tables

**Table 1:** Systematic-lupus-erythematosus-associated cerebellar progressive multifocal leukoencephalopathy.

Age/Sex	Past History Other than SLE	Steroid (mg/day)	Immunosuppressant	Specimen/JCV Copy Number (Copies/mL)	Treatment			JCV-DNA Copy Number Trends	Follow-up Period/Prognosis	References
					Meflo- quine	Mirtaz- apine	Other			
50/F	Lymphoma	PSL/5	MMF, RIT, Bendamustine	CSF, brain biopsy	+	+	None	N.A.	5 m/dead	Sakuraba [19]
68/M	None	N.A.	TAC, MTX	CSF	+	+	None	Neg/6 m	9 m/alive	Hamaguchi [16]
60/F	None	PSL/12	MTX	CSF/67,000	−	−	N.A.	N.A.	3 m/dead	Zhong [38]
27/F	RA	PSL/10–20	ETN, TOC, RIT, MTX	CSF	+	−	DFPP, Paroxetine	Neg/4 m	12 m/alive	Cheng [14]
34/F	None	Deflazacort/6	AZA	CSF/700	+	+	CDV, Ara-C	Increased/6 m	12 m /dead	Berntsson [12]
32/F	HPS	N.A.	CPA, AZA, MTX, RIT, Thalidomide	CSF	−	−	CDV	N.A.	N.A.	Brandão [13]
23/F	RA	PSL/10–20	Hydroxychloroquine, ETN	CSF/28,600	−	−	Ara-C, PE	N.A.	N.A./dead	Graff-Radford [15]
51/M	N.A.	N.A.	N.A.	Biopsy	N.A.	N.A.	N.A.	N.A.	36 m/alive	Svensson [37]
36/F	None	PSL N.A.	CYA	CSF	−	−	CDV	Neg/74 days	7 m/dead	Salmaggi [20]
21/F	None	PSL/30	None	Autopsy	−	−	Interferon	N.A.	23 m/dead	Kinoshita [18]
55/F	None	PSL/15	None	Biopsy	−	−	None	N.A.	3 m/dead	Jones [17]
57/F	None	PSL/5	MMF	CSF/15,8700	+	+	None	Neg/26 m	27 m/alive	Our case

Ara-C, cytarabine; AZA, azathioprine; CDV, cidofovir; CPA, cyclophosphamide; CYA, cyclosporine; CSF, cerebrospinal fluid; DFPP, double filtration plasmapheresis; ETN, etanercept; F, female; m, months; M, male; HPS, hemophagocytic syndrome; JCV, John Cunningham virus; MMF, mycophenolate mofetil; MTX, methotrexate; N.A., not available; Neg, negative; PE, plasma exchange; PML, progressive multifocal leukoencephalopathy; PSL, prednisolone; RA, rheumatoid arthritis; RIT, rituximab; SLE, systematic lupus erythematosus; TAC, tacrolimus; TOC, tocilizumab.

## Data Availability

Not applicable.

## Supplementary Materials

The following supporting information can be downloaded at: https://www.mdpi.com/article/10.3390/neurolint15030059/s1. Figure S1: The result of an in-house tissue-based assay.
