# Supplementary material for: Cerebellar Progressive Multifocal Leukoencephalopathy Mimicking Anti-Yo-Antibody-Associated Rapidly Progressive Cerebellar Syndrome
Source: Neurol Int. 2023 Jul 26;15(3):917–25. doi: 10.3390/neurolint15030059 (PMC10443244; doi:10.3390/neurolint15030059)
Supplement: Supplementary file 1 [file neurolint-15-00059-s001.zip › neurolint-2451391-supplementary.pdf]

Supplementary Figure

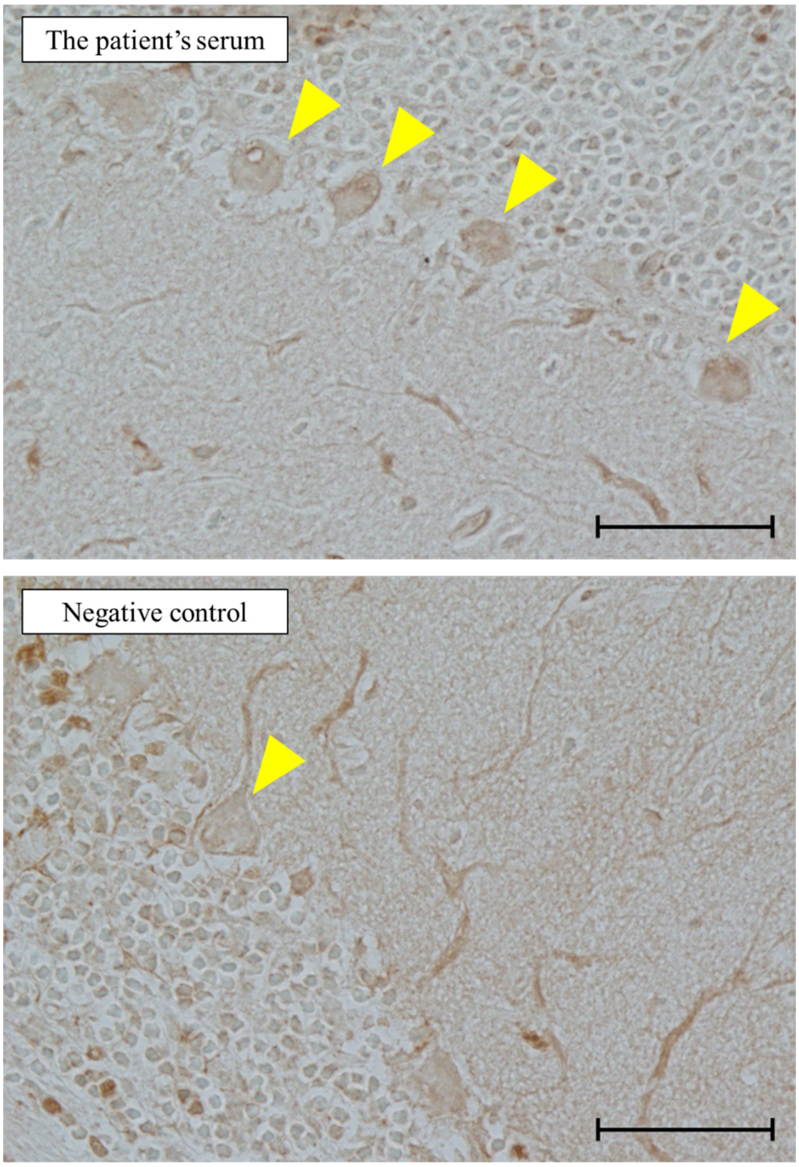

Figure S1. In-house tissue-based assay was performed as follows. An adult female Wistar rat was sacrificed without perfusion. The brain was removed and fixed in 4% paraformaldehyde for 1 hour at 4°C, cryoprotected in 40% sucrose for 48 hours, embedded in freezing compound media, and snap frozen in isopentane chilled with liquid nitrogen. Seven-micrometer-thick tissue sections were then sequentially incubated with 0.3% H2O2 for 15 minutes, 5% goat serum for 1 hour, and patient or control serum at 4°C overnight. After incubating with biotinylated Ig-class-specific secondary antibodies against human IgG (1:2000, BA-3000, Vector), the reactivity was developed with the avidin-biotin-peroxidase method. The pictures show the Purkinje cell layer and granule cell layer of rat cerebellum. Sections reacted with patient serum showed no staining to Purkinje cells, as did negative controls (yellow allow head). If anti-Yo antibody would be present in the patient's serum, the Purkinje cells would be labeled. Scale bar = 50 μm.
